# Inclusion Complex of Resveratrol with γ-Cyclodextrin as a Functional Ingredient for Lemon Juices

**DOI:** 10.3390/foods10010016

**Published:** 2020-12-23

**Authors:** Andreia F. R. Silva, Mariana Monteiro, Daniela Resende, Susana S. Braga, Manuel A. Coimbra, Artur M. S. Silva, Susana M. Cardoso

**Affiliations:** LAQV-REQUIMTE, Department of Chemistry, University of Aveiro, 3810-193 Aveiro, Portugal; afrs@ua.pt (A.F.R.S.); marianaicnamonteiro@gmail.com (M.M.); danielaresende@outlook.com (D.R.); sbraga@ua.pt (S.S.B.); mac@ua.pt (M.A.C.); artur.silva@ua.pt (A.M.S.S.)

**Keywords:** cyclodextrin, inclusion, resveratrol, solubility, stability, lemon juice, antioxidant, beverage, citric acid

## Abstract

Microencapsulated resveratrol (RSV) is a pertinent ingredient in functional foods to be used in the prevention and management of cardiovascular diseases. Gamma-cyclodextrin (γ-CD) was evaluated for its RSV inclusion ability. Inclusion procedures comprised mixing equal concentration of an aqueous solution of γ-CD with an ethanol solution of RSV and freeze-drying to obtain a solid material. Solid-state characterization by vibrational spectroscopy, thermogravimetry, and powder X-ray diffraction (PXRD) confirmed the formation of the γ-CD·RSV complex in a ratio of 1:1. PXRD suggested that cyclodextrin molecules in the complex are stacked in infinite channels holding the RSV inside, with a wide inter-channel space where 14 water molecules are retained. Fresh lemon juices supplemented with 0.625 mg/mL of RSV in its free (RSV-juice) or complexed (γ-CD·RSV-juice) form were stored along 28 days under dark and room temperature or at 4 °C. Initially, the RSV level in γ-CD·RSV-juice was about nine times higher than in RSV-juice (43.1% and 4.8%, respectively), suggesting that the RSV complexation promoted its solubility in the lemon juice, a fact that was still noticed after 28 days of storage. Moreover, regardless the fact that the antioxidant capacity was similar among the juices, the loss of antiradical ABTS^•+^ capacity in γ-CD·RSV-juice was reduced compared to that of the RSV-juice. Overall, this study allowed concluding that γ-CD can serve as a carrier of RSV, promoting its solubility and eventually protecting its antioxidant stability in lemon juices for at least 28 days.

## 1. Introduction

Resveratrol (RSV) is a natural secondary metabolite of the family of stilbenes that is quite valuable as a functional ingredient due to its pleiotropic health beneficial effects as anti-inflammatory, antitumoral, and protecting action on the cardiovascular system [1,2,3]. The bioactive form of this compound is the *trans*-isomer, which in nature can be found preferentially in white hellebore roots (*Veratrum grandiflorum* O. Loes) [4], Itadori plant (*Polygonum cuspidatum*), grapes and wine (*Vinis vinifera*), and peanuts [5,6]. Once these natural sources of RSV are not usually consumed in every diet, the supplementation of products of large consumption with RSV is a good strategy to improve the daily intake of this compound. However, the lipophilicity of RSV and its sensitivity to external factors such as light, air exposure and oxidative enzymes, are limiting factors to its stability in aqueous media, as well as to its bioavailability and use in the preparation of functional foods [7].

A growing technological solution in food supplementation is the use of native cyclodextrins (CDs), naturally occurring cyclic oligosaccharides of 1,4-linked α-d-glucose formed by bacterial degradation of starch through the enzyme cyclodextrin glycosyl transferase [8]. The most abundant and affordable native CDs occur with six to eight glucose units and receive the names of α-CD, β-CD and γ-CD, respectively. CDs are approved for use as food additives by the US Food and Drug Administration (FDA) and the World-Health Organization-Food and Agriculture Organization of the United Nations (WHO-FAO) joint committee, having the GRAS status (FDA list of food additives that are generally recognized as safe). Owing to their ring-shaped structure, CDs act as inclusion hosts for a variety of compounds, such as RSV. The resulting inclusion complexes have typically increased aqueous solubility and the included guests are protected from aggressive external conditions such as oxidation and degradation by UV radiation or heat [9].

Resveratrol is reported to interact with a variety of cyclodextrins, with improvements not only to its solubility but also its photostability and activity [10,11,12,13,14]. The types of interactions formed, however, depend on the host cavity size, with α-CD, the narrowest of native cyclodextrins, being reported as a good solubilizer for RSV in aqueous media [15,16] but as unable to form authentic inclusion complexes with this guest in the solid-state [17]. β-CD has a less pronounced solubility enhancement effect on RSV [15,16], but, owing to its larger cavity, it forms with this guest a co-amorphous solid that can be used as a safe oral delivery system (no hematologic alterations were observed in a mouse model at a dose of 1 mg/kg of the compound) [18]. γ-CD, having the largest cavity size among the three native CDs, can fully accommodate RSV inside it to form authentic inclusion complexes. The crystal structure of (γ-CD)_3_·(RSV)_4_·(H_2_O)_62_ was reported earlier this year, the host molecules stacked into channels in which the disordered RSV molecules are included [17]. Interestingly, the solubilizing effect of γ-CD on RSV is similar to that exhibited by α-CD [15,16]. RSV is also described to form inclusion complexes with several chemically modified CDs, including the randomly methylated derivatives of α-CD (RAMEA) [19] and of β-CD (RAMEB) [19,20], the 2,6-permethylated derivative of β-CD (DIMEB) [19], sulfobutylether-β-CD [21] and the randomly (2-hydroxy)propylated derivative of β-CD (HP-β-CD) [10,11,12,22,23]. HP-β-CD, in a comparative study, was demonstrated to have the strongest binding affinity to RSV, followed by RAMEB [24]. The hosts β-CD and HP-β-CD are also reported to include oxyresveratrol, an oxidized derivative of RSV, forming inclusion complexes that are suitable delivery systems for food applications. The HP-β-CD inclusion complex with oxyresveratrol brings strong antibrowning effects on fresh grape juice [25], while milk and orange juices models fortified with β-CD·oxyresveratrol were reported to remain stable over five weeks [26]. Moreover, the complex features increased dissolution rate for resveratrol, as well as improved antioxidant activity and amounts of bioaccessible oxyresveratrol in in vitro digestion assays.

Among native CDs, γ-CD is considered the most suitable for food applications due to its lower toxicity (lethal dose, 50% > 8000 mg/kg body weight for oral administration in rat) and to the fact that it is the only native CD that undergoes complete digestion in the gastrointestinal tract [27]. There are already a few reports on the formation and use as food fortificants of γ-CD inclusion complexes with bioactive components such as thymol [28], gingerols [29] and quercetin [30]. While the scarce number of reported studies of complexes with γ-CD in food matrices reflects the slightly high cost of this host, in comparison with β-CD (the most affordable cyclodextrin available), native CDs are considerably more appealing than the modified ones (e.g., the price per ton of HP-β-CD may be from 50 to 200 times that of β-CD). Moreover, relevant for application in liquid foods is the fact that pure aqueous solutions of γ-CD have the particularly of quickly becoming opalescence upon storage, with small visible precipitates [27]. Opalescence may limit the application of γ-CD in some foods but, at the same time, it may be an attractive characteristic in formulations of opaque juices, similar to the “homemade” ones, which represents a new market trend.

Beverages are a type of product that has been widely involved in the scope of functional foods, with great notoriety of probiotic dairy-based beverages, energy drinks, and enriched vegetable and fruit-based beverages [31]. On the other hand, the juice is a product of large consumption, advantageous to be a vehicle of RSV to daily diet toward the prevention of diseases such as cardiovascular disorders. In the present work, the formation of a γ-CD complex with RSV was studied, followed by supplementation of the obtained γ-CD·RSV complex in natural lemon juices as a model for liquid foodstuff. The aim was to investigate the impact of γ-CD complex on *trans*-RSV stability and antioxidant activity when integrated into lemon juices under storage conditions. Overall, we expect to provide new information on the application of γ-CD·RSV complex in foodstuff, a topic that remains largely unexplored.

## 2. Materials and Methods

### 2.1. Chemicals

γ-Cyclodextrin (γ-CD), with a water content of ca. 9%, was manufactured by Wacker under the tradename Cavamax W8 and kindly donated by Ashland Industries Deutschland Gmbh (Düsseldorf, Germany). Resveratrol (RSV) was purchased from Fluorochem (Derbyshire, United Kingdom). Ethanol and citric acid were purchased from Panreac (Barcelona, Spain). 2,2′-Azino-bis(3-ethylbenzothiazoline-6-sulphonic acid) (ABTS), benzoic acid, trolox, phenazine methosulfate, β-nicotinamide adenine dinucleotide (β-NADH), nitrotetrazolium blue chloride (NBT), phenazine methosulfate (PMS), and potassium bromide were obtained from Sigma-Aldrich (St. Louis, MO, USA).

### 2.2. Preparation of the Inclusion Complex (γ-CD·RSV) and Physical Mixture

Inclusion of RSV into γ-CD was achieved by mixing two solutions of the individual components. An aqueous solution of γ-CD was prepared by stirring 3.11 g (0.3 mmoL) in 146.2 mL of ultrapure water at 40 °C. After the complete dissolution of γ-CD, the aqueous medium was treated with an ethanolic solution of 500 mg (0.3 mmoL) of RSV in 73.1 mL of absolute ethanol and stirred for homogenization. The mixed solution was then subject to snap-freezing by immersion in liquid nitrogen and freeze-dried for two days. The resulting solid product was collected into a glass vial and allowed to rehydrate in a water-saturated chamber for 24 h.

The physical mixture (1:1) of RSV and γ-CD was prepared by gently mixing equimolar amounts of the two powders with a spatula.

### 2.3. Characterisation of the Inclusion Complexes

#### 2.3.1. Infrared Spectroscopy

RSV, γ-CD, γ-CD·RSV complex and physical mixture were subjected to Fourier-transform infrared (FT-IR) spectroscopic studies in a GALAXY Series FT-IR 7000 Spectrophotometer (Mattson Instruments, Wellesley, MA, USA), resolution 2 cm^−1^, averaging 64 scans per sample in the region of 4000 to 300 cm^−1^. Samples were mixed in a mortar with potassium bromide (1:100) and pressed in a hydraulic press (9 tons) to small pellets, which were then placed in the infrared beam.

#### 2.3.2. Powder X-ray diffraction (PXRD)

Laboratory PXRD data were collected at ambient temperature on an X’Pert MPD Philips diffractometer (Cu, Kα1 = 1.540598 Å) (Bruker AXS, Karlsruhe, Germany) with a curved graphite monochromator, equipped with a X’Celerator detector (Bruker AXS, Karlsruhe, Germany), operating in a flat Bragg-Brentano configuration (40 kV, 50 mA). Data were collected with steps of 0.04° in a continuous mode in the 3.5° ≤ 2θ ≤ 50° interval.

#### 2.3.3. Thermogravimetric Analysis (TGA)

TGA studies were performed on a Shimadzu TGA-50 thermogravimetric analyzer, using a heating rate of 5 °C min^−1^, under air atmosphere, with a flow rate of 20 mL/min. The sample holder was a 5 mm Ø platinum plate, and the sample mass was about 5 mg. 

### 2.4. Formulation of Lemon Juices

Firstly, 40 mL of natural lemon juices, i.e., squeezed from fresh lemons, were supplemented with either 25 mg (10.9 × 10*^−^*^5^ moles) of pure *trans*-RSV (RSV-juice) or 194.6 mg of γ-CD·RSV inclusion complex (γ-CD·RSV-juice), i.e., equivalent to 0.625 mg/mL. Then, solutions of citric acid (CA), also with the volume of 40 mL and at a CA concentration of 0.18 M, were supplemented with the same amount of *trans*-RSV (RSV-CA) or with γ-CD·RSV (γ-CD·RSV-CA) and used as controls. Finally, 0.1 mg/mL of benzoic acid was added to the juices as a preservative to approach the hypothetical conditions of a natural lemon juice suitable for the market. Samples were stored in the dark for 28 days at two different temperatures: 4 °C and room temperature.

### 2.5. Physicochemical Analysis of the Liquid Samples

The pH of the samples was measured at room temperature with an adequately calibrated electrode (pH electrode PC52+DHS, XS Instruments, Carpi Mo, Italy).

The following analyses were performed based on the procedures described by Queirós et al. [32]: browning degree value was determined after centrifugation of the juice samples at 8964 rpm for 20 min by reading the absorbance of the supernatant at 420 nm in a UV–Vis microplate multimode reader (Synergy|HTX, Bio Tek, Winooski, VT, USA); cloudiness was evaluated by absorbance measurement at 700 nm using the same equipment; the °Brix was determined with a handheld refractometer (ATC-1E, Atago, Bellevue, WA, USA) at room temperature.

Total color difference (ΔE) was measured through colorimetric CIElab parameters (L*a*b*) using a colorimeter (CM 2300d, Konica Minolta, Tokyo, Japan).

### 2.6. Stability Studies

The stability of formulated juices over the time of storage was monitored regularly at prescribed time points. This included a zero-time point (i.e., just after preparation, t0) and measurements at 7, 14, 21, and 28 days. Measurements included the determination of RSV in solution and of the antioxidant capacity. Before analysis, 2 mL of formulated juices were collected, centrifuged at 10,500 rpm for 5 min and filtered through a filter paper. Afterward, 300 µL of the supernatant was resuspended with 700 µL of absolute ethanol to disintegrate the γ-CD·RSV complex and guarantee RSV release and total solubility, followed by filtration throughout a 0.22 µm nylon filter (Whatman™). The choice of 70% of ethanol as a solvent for total RSV solubilization was based on preliminary results obtained in our lab.

#### 2.6.1. Quantification of RSV

The concentration of RSV in the liquid samples was determined at 306 nm by ultra-high-performance liquid chromatography UHPLC. Equipment setup comprised a quaternary pump, an autosampler, an ultimate 3000 Diode Array Detector (Dionex Co., Sunnyvale, CA, USA) and an automatic thermostatic column compartment. An end-capped Hypersil Gold C18 column (Thermo Scientific, Waltham, MA, USA) with 100 mm length and 2.1 mm i.d. was filled with particles of 1.9 μm Ø and kept at a temperature of 30 °C. Gradient elution was carried out with aqueous 0.1% of formic acid (*v*/*v*) (solvent A) and acetonitrile: methanol (70/30) (solvent B). The solvent gradient consisted of a series of linear gradients, starting with 27% of solvent B over 4 min, increasing to 50% over 5 min and to 100% over 15 min, maintaining this value up to 25 min, followed by the return to the initial conditions, with a total running time of 35 min. The flow rate used was 0.2 mL/min. *trans*-RSV calibration curve was performed for 11–108 μg/mL, using 70% ethanol as a solvent for RSV.

#### 2.6.2. Antioxidant Activity

The antioxidant activity of juices was evaluated by measuring their ability to scavenge the ABTS^●+^ and the SO^●+^ radicals.

The ABTS scavenging activity was determined by the method of Saada et al. [33]. Trolox (between 0 and 0.2 mM) was used as a positive control and in the calibration curve. In the procedure, 50 μL of juice/standard were added to 250 µL of diluted ABTS^●+^ solution. The mixture was incubated for 20 min under dark conditions and the absorption at 734 nm was subsequently measured using a microplate multimode reader (Synergy|HTX, Bio Tek, Winooski, VT, USA). The results were expressed as mM trolox equivalents (mM TE).

Radical-scavenging ability towards SO^●+^ was measure according to Pereira et al. [34], also using trolox as a positive control and a calibration curve (concentrations between 2.8 and 20 mM). The procedure required the addition of 75 μL of NBT, 100 μL of NADH, 50 μL of PMS, and 75 μL of trolox standard/juice samples, followed by 5 min of incubation and absorbance measurement at 560 nm. The results were expressed as M trolox equivalents (M TE).

### 2.7. Statistical Analysis

Statistical analysis of data (at least from three independent assays) was performed on a trial version of GraphPad Prism 6.01 software (OriginLab Corporation, Northampton, MA, USA) using one and two-way ANOVA analysis by Tukey’s multiple comparisons test for comparison of individual means. In all cases, the significance level was set as *p* < 0.05.

## 3. Results and Discussion

### 3.1. Preparation and Solid-State Characterisation of the γ-CD Inclusion Complex with RSV

The good aqueous solubility of the host, γ-CD, allows using water as a solvent, while RSV required a less polar solvent to dissolve. Given the envisioned application in foodstuff, and considering the adequate solubility of RSV in ethanol, this was elected as a biocompatible organic solvent for RSV. The two solutions were mixed to obtain a pristine clear mixed solution where γ-CD and RSV interacted to form an inclusion complex. Thus, the co-dissolution method was efficient to form the inclusion of the RSV guest molecule into γ-CD, in a ratio of 1:1.

#### 3.1.1. FT-IR

**Figure 1:**
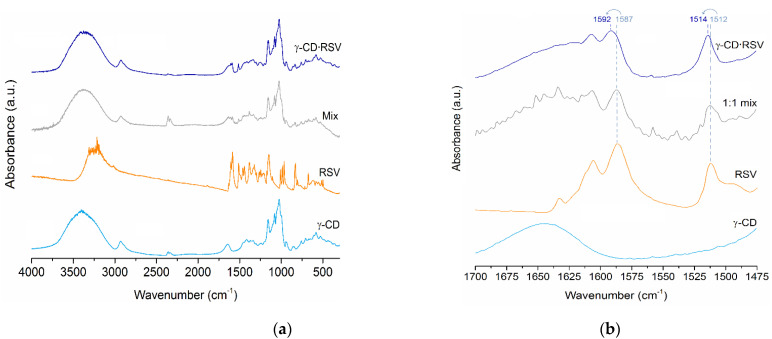
(**a**) FTIR spectra of gamma-cyclodextrin (γ-CD) (light blue), resveratrol (RSV) (orange), physical mixture (Mix, grey) and γ-CD·RSV hydrated complex (dark blue). The inset, (**b**), represents a selected spectral window where some guest bands are visible in γ-CD·RSV (dark blue), with highlight to those at 1592 and 1514 cm^−1^ that are blueshifted in regard to the same bands of pure RSV (orange) and 1:1 mix (grey), occurring at 1587 and 1512 cm^−1^; γ-CD (light blue).

Analysis of the solid product of the inclusion procedures by FT-IR spectra is a quick and insightful tool to confirm the formation of an inclusion complex by observation of alterations in relevant guest bands sensitive to the hydrophobic cavity of γ-CD; that is, oscillators that can act as inclusion probes. FT-IR spectra of RSV, γ-CD, γ-CD·RSV complex (hydrated) and the 1:1 physical mixture of γ-CD and RSV (Mix) are depicted in Figure 1a.

The spectrum of RSV shows various characteristic peaks according to literature reports [17,21,35]: the free O–H stretching vibration peaks, resulting in a broad band at 3250 cm^−1^ (arising from water molecules in the normal atmosphere), the C=C aromatic stretching is observed at 1606 cm^−1^, the olefinic C–C stretching vibration is observed at 1587 cm^−1^, and the C=C–H bending vibrations at 965 and 988 cm^−1^.

The spectrum of γ-CD·RSV is in good agreement with the one recently reported by Catenacci et al. [17] for the same inclusion complex prepared by kneading. The spectrum is dominated by a few intense host bands, namely, the O–H stretch, peaking at 3370 cm^−1^, the C–H stretch, peaking at 2900 cm^−1^, and the C–O stretch at 1028 cm^−1^. Guest bands can also be observed in the 1700 and 1475 cm^−1^ region, in particular the C=C stretching band at 1606 cm^−1^, with an intensity matching of the same band in the spectrum of the 1:1 physical mixture, thus indicating that the stoichiometry was retained during the inclusion procedures. Most importantly, the νC=C band occurs at 1592 cm^−1^, which represents a blueshift of 5 cm^−1^ in regard to pure RSV and the physical mixture, confirming the formation of an authentic inclusion complex by the herein employed freeze-drying method. The increased energy of a stretching vibration can be explained by the restrictions in molecular motion associated with the confinement of the RSV molecule into the cavity of γ-CD. Moreover, a blueshift of 5 cm^−1^ for the same band was also reported by Catenacci et al. [17] for their kneaded inclusion complex of RSV with γ-CD. Another band, at 1514 cm^−1^, also appears slightly blueshifted by 2 cm^−1^ in the spectrum of γ-CD·RSV (Figure 1b), but this value is within the spectral resolution. In the FT-IR spectrum of the physical mixture, it was possible to observe peak contributions from both RSV and γ-CD, but with no significant shifts (Figure 1a), indicating that RSV did not interact with γ-CD.

#### 3.1.2. PXRD

**Figure 2:**
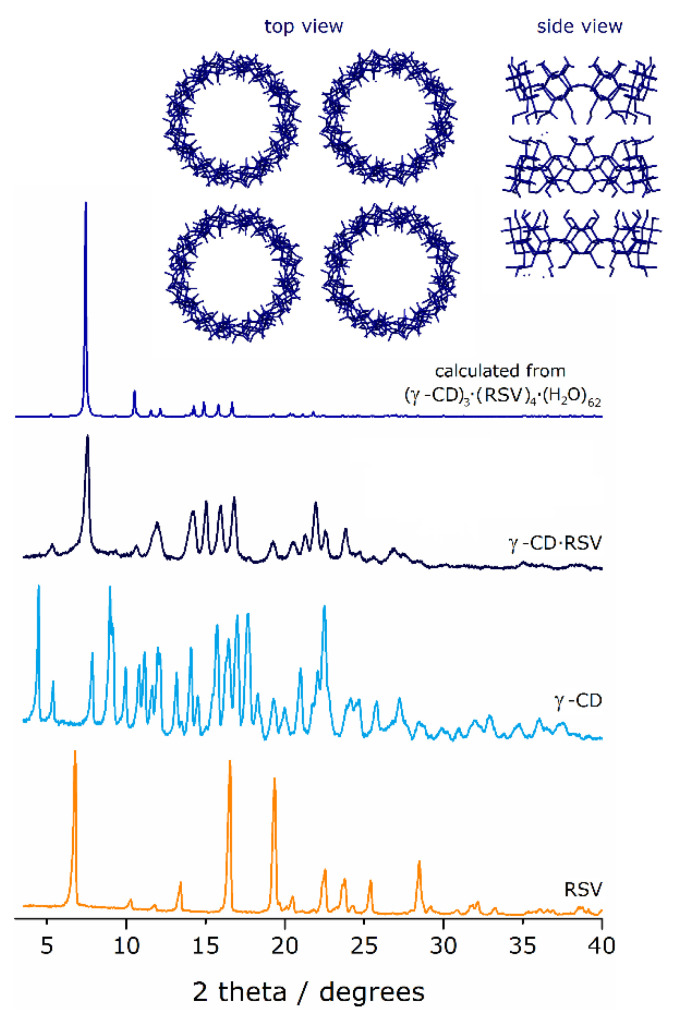
Experimental powder X-ray diffractograms (PXRD) of RSV, γ-CD, and the γ-CD·RSV complex (rehydrated overnight to restore its microcrystalline structure); also shown for comparison is the trace of (γ-CD)_3_·(RSV)_4_·(H_2_O)_62_, calculated from its single-crystal atomic coordinates [17]. Structural data for this complex (refcode MUXBIT) was obtained from CCDC and the PXRD was calculated using the software Mercury 3.5.1 (Copyright CCDC 2001–2014). The inset depicts the top and side views of the complex, with γ-CD units aligned in the form of channels (disordered RSV not shown).

PXRD diffraction patterns of RSV, γ-CD, and γ-CD·RSV complex were collected and analyzed to investigate complex formation (Figure 2).

No peaks associated with the pure compounds γ-CD and RSV (for example, 4.5, 8, 28 to 40 degrees of γ-CD and 19, 28–40 degrees of RSV) are visible in the diffractogram of γ-CD·RSV, which indicates the formation of a new phase associated with the inclusion complex of RSV with γ-CD. Moreover, the peak profile of the new phase can be identified as belonging to the isostructural series described by Caira for tetragonal, channel-packed γ-CD inclusion complexes with a variety of guest [36]. For further confirmation, the trace for the reported complex (γ-CD)_3_·(RSV)_4_·(H_2_O)_62_ was calculated from its single-crystal structural data and it is depicted for comparison, showing fair similarities. It can thus be inferred that the γ-CD·RSV complex herein reported also features channel packing.

#### 3.1.3. TGA

**Figure 3:**
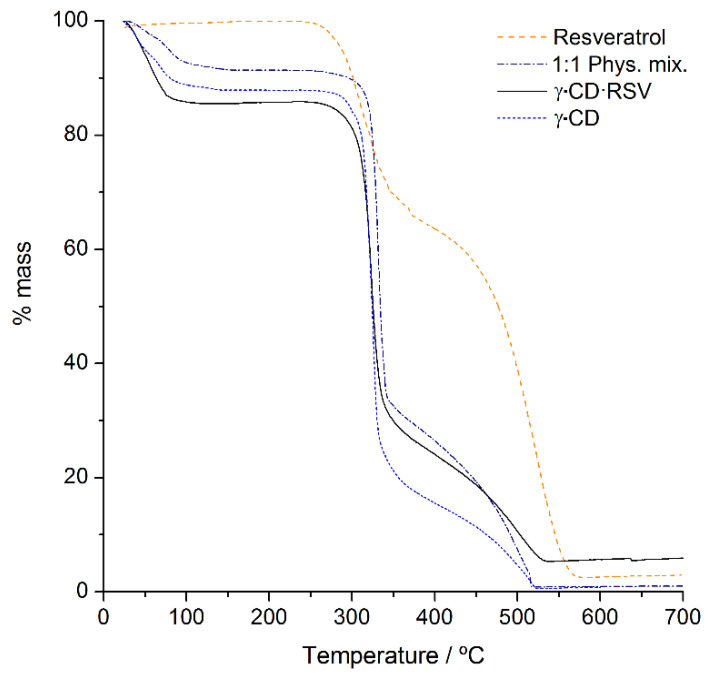
Thermogravimetric traces of RSV (dashed line), γ-CD·RSV (solid line), the 1:1 physical mixture of γ-CD with RSV (dash-dot line), and γ-CD (short-dash line).

TGA was employed to investigate the relationship between temperature change and weight loss of the complex, providing information regarding its thermal stability and initial composition. It was observed that RSV is quite thermally stable, as it starts to decompose only around 250 °C (Figure 3). The absence of mass losses between room temperature and 130 °C (temperature interval associated with dehydration) denotes that RSV contains no hydration water molecules, confirming its hydrophobic nature. In the case of γ-CD and its products, there are some similarities in decomposition temperature (lines of γ-CD, γ-CD·RSV and 1:1 phys. mix, Figure 3), as well as the expectable presence of hydration water molecules.

Thermal decomposition profile of γ-CD occurs in two major mass losses, one initial step up to 100 °C due to dehydration, followed by a plateau and then a second step ascribed to decomposition that starts around 265 °C and proceeds up to 500 °C [29]. The dehydration steps (i.e., the difference between the first peak of mass and the first plateau) featured mass losses of 8% for the physical mixture, 12% for pure γ-CD hydrate, and 14.5% for the complex. The lower percentage of water in the mixture regarding pure γ-CD is probably due to the presence of the non-hydrated RSV in its composition. In turn, the increase in the amount of hydration water molecules is a typical feature of inclusion complexes that have γ-CD as the host. It results from the tetragonal geometry. When viewing the channels from the top, one can easily perceive that their centers are aligned to form squares, as seen in the inset of Figure 2, which leaves large void inter-channel spaces that can accommodate a large number of water molecules. Thus, the mass loss of 14.5% in the γ-CD·RSV complex corresponded to the presence of 14 molecules of water per complex unit, enabling the deduction of the composition of the complex as γ-CD·RSV·(H_2_O)_14_. Notably, in the fully crystalline form of the complex reported by Catenacci et al. [17], (γ-CD)_3_·(RSV)_4_·(H_2_O)_62_, the number of hydration waters is even higher, at an average of 20.6 water molecules per γ-CD molecule.

### 3.2. Stability of RSV in Formulated Juices

Natural lemon juices were supplemented with free RSV (RSV-juice) and with the complex of γ-CD·RSV (γ-CD·RSV-juice) and their physicochemical parameters are represented in Table 1. Based on the literature [37], solutions of citric acid (0.18 M) with free RSV (RSV-CA) and with complexed RSV (γ-CD·RSV-CA), with the same pH of lemon juices, i.e., close to 2.5, were prepared and used as control models to simulate the conditions of lemon juice matrices. According to the results, the appearance of juices was slightly yellowish and cloudy. This characteristic constitutes an important barrier against microbial growth, and it facilitates its preservative conditions. The °Brix (related to the content in total soluble solids) was 8.7 and 9.0 in RSV-juice and γ-CD·RSV-juice, respectively. This value was concordant with FAO regulations that stipulated a minimum of 8.0 for °Brix of lemon juices (from *Citrus limon* Burm. f.) for nectars purposes [38]. Moreover, both pH and °Brix of the lemon juices were similar to the isotonic beverages enriched with lemon juices reported by Gironés-Vilaplana et al. [39]. There are no natural fruit sugars among the soluble solids for the controls (i.e., citric acid solutions), thus justifying the low °Brix of 3.5.

Lemon juices and CA also clearly differed (*p* > 0.05) with respect to their browning, with values of approximately 0.20 and 0.04, respectively. The browning degree of the juices (0.19 and 0.20) could be related to their higher content in ascorbic acid and other compounds, which can be mediated by degradation in strong acid conditions, generate browning compounds in solution [40]. Furthermore, the degree of cloudiness seems to vary among samples, with higher values reported in those supplemented with γ-CD·RSV, particularly in CA solutions. Considering the current tendency in the market to have natural juices presenting a cloudier appearance, this could be an interesting feature for consumer’s acceptability.

All the above-mentioned characteristics influence the final color of juices. Once juices were neither transparent nor opaque, but translucent, we opted by comparing the formulated samples according to their ΔE, using lemon juice as reference (Table 1). The values of ΔE were used as guidelines to classify color differences in not noticeable (0–0.5), slightly noticeable (0.5–1.5), and noticeable (1.5–3.0), or even well visible (3.0–6.0) and great visible (6.0–12.0) [41]. In this work, a ΔE close to six was found between formulated juices and plain lemon juice, meaning that the color change was visible. This is a relevant aspect to consider regarding the consumer’s acceptability in case of future juice formulations.

#### 3.2.1. RSV Integrated in Juice Matrix

**Figure 4:**
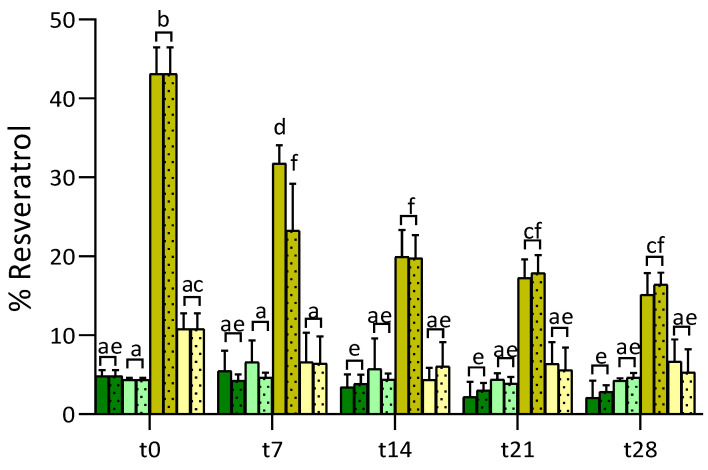
Percentage of resveratrol dissolved in RSV-juice (dark green), RSV-CA (light green), γ-CD·RSV-juices (yellowish green) and γ-CD·RSV-CA (light yellow) along 28 storage days at room temperature (rt) (without pattern) and 4 °C (dotted pattern). Different letters in the same column indicate significant differences (*p* < 0.05).

The percentage of RSV dissolved in the fortified juices and CA solutions over 28 days of storage at 4 °C or room temperature is represented in Figure 4. 

Immediately after formulation (t0), RSV levels in the RSV-fortified samples (i.e., RSV-juice and RSV-CA) corresponded to less than 5% of the target concentration (0.625 mg/mL), while values in γ-CD·RSV-fortified samples were significantly higher, namely of 45.8% in γ-CD·RSV-juice and 9.1% in γ-CD·RSV-CA (corresponded to 0.286 and 0.057 mg of RSV mL^−1^ juice, respectively). These results suggest that the presence of CD improved the dissolution of RSV. The same trend was observed by He et al. [25] for the oxyresveratrol-β-CD complex, which allowed a 100% dissolution rate of RSV just after 10 min, comparatively to 64.5% of free RSV after 2 h of dissolution.

Over storage time, the percentage of dissolved RSV in juice samples (RSV-juice and γ-CD·RSV-juices) tended to decrease. In γ-CD·RSV-juices, it was reduced to half of the initial value between t14-t21 (from 43.1% to 19.8%), which may suggest some disintegration/degradation of the complex in this period of time. This result was less promising than that reported by He et al. [25] for Oxy-β-CD and Oxy-HP-β-CD. Their report showed that complexes remained relatively stable in aqueous media and at 25 °C in the dark, holding more than 60% of oxyresveratrol after 30 days. To our knowledge, the stability of γ-CD·RSV complex in aqueous solutions at pH 2.5 (i.e., equivalent to juice pH) was not studied yet. Nevertheless, we can assume that the affinity between RSV and γ-CD at this pH value is identical to the one measured in ultrapure water (inclusion constant, K_1:1_ = 224 M^−1^) [24], since in both cases, RSV is in its neutral form. Thus, our results can be interpreted as resulting from other phenomena, such as competing interactions of γ-CD with other juice components like terpenes. The contribution of competitive inclusion towards partial complex degradation requires additional studies.

Compared to γ-CD·RSV-juice, levels of RSV in γ-CD·RSV-CA were lower (45.8% vs. 9.1% at t0), which is probably due to their composition (multiple components in juice vs. citric acid). Moreover, one should not discard the fact that the theoretical citric acid concentration herein used (0.18 M) does not exactly correspond to that of the juices (not determined) and that this may also interfere in complex solubilization. In fact, citric acid is known to act as a solubility enhancer for β-CD, probably by interfering with its intramolecular homodromic hydrogen bridge chain [42]. Based on this, one may hypothesize that CD destabilizes the γ-CD·RSV complex either by disruption of its supramolecular structure (held together by hydrogen bonds) or even by direct competition between CA and RSV for inclusion into the cavity of the γ-CD. Such destabilization of γ-CD·RSV would afford free, insoluble RSV, which would be dispersed in the juice in a non-quantifiable manner. This effect may be less noticeable in natural juices due to the presence of other natural compounds and/or lower amounts of citric acid.

Overall levels of dissolved RSV in the CA solutions (RSV-CA) did not vary along the storage period. Moreover, the tendency to reduce the dissolved RSV levels after 7 days of storage, observed in γ-CD·RSV-CA at both storage temperatures, was not statistically significant. Thus, the complex γ-CD·RSV was the best candidate for a functional ingredient of lemon juice.

#### 3.2.2. Antioxidant Activity

**Figure 5:**
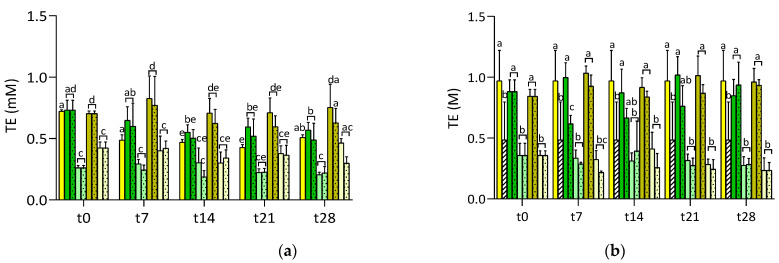
Radical-scavenging ability of ABTS^•+^ (**a**) and SO^•+^ (**b**) of formulated juices in RSV-juice (dark green), RSV-CA (light green), γ-CD·RSV-juices (yellowness green) and γ-CD·RSV-CA (light yellow) along time of storage, protected from light, at room temperature (rt) (without pattern) and 4 °C (pattern dots). Plain lemon juice at room temperature (yellow without pattern) and citric acid solution at room temperature (0.18 M, white with striped pattern) are represented as reference. Different letters in the same column indicate significant differences (*p* < 0.05).

The ability of the samples to scavenge ABTS^•+^ and SO^•+^ over the 28 days of storage at room temperature and 4 °C is shown in Figure 5. No differences were observed between the two fortified juices (RSV-juices and γ-CD·RSV-juices) or between fortified-juices and the plain lemon juice, regardless of their distinct RSV amounts. At t0, the ABTS^•+^ scavenging ability of γ-CD·RSV-juices and RSV-juices were 0.75 mM and 0.70 mM TE, respectively (Figure 5a), and those of SO^•+^ scavenging were both close to 0.7 M of TE (Figure 5b). Moreover, albeit γ-CD·RSV-CA tended to be more active against ABTS^•+^ than RSV-CA, no significant difference was observed between these two samples. Moreover, they exhibited a similar ability to scavenge SO^•+^, which was close to that of a plain CA solution. Hence, the gathered results suggest that the antiradical ability of RSV in the fortified samples was masked by the high antioxidant capacity of the plain juices/CA, which in turn is most probably due to the high amounts of CA, and, in the case of lemon juice, to its combined richness in CA and other antioxidants like vitamin C and phenolic compounds. However, it is worth highlighting the work of Anselmi et al. [43], as they concluded that independently of inclusion phenomena, some chemical groups of bioactive compounds that are involved in free radical quenching tend to interact with the hydroxyl groups of cyclodextrin, being thus less available to interact with radicals and to exert their activity.

In the literature, small differences in scavenging activity were observed by Duarte et al. [20] between free RSV and the inclusion complex of RSV with a biocompatible variety of randomly methylated β-CD (CRYSMEB), which also featured increased solubility. In turn, Lu et al. [12] reported a superior DPPH^•^ scavenging activity for β-CD·RSV complex in comparison to RSV.

The antiradical ABTS^•+^ capacity of plain lemon juice decreased significantly over storage, by up to 32.4% at t7 and 34.8% at t14, probably denoting the degradation of antioxidant compounds. Losses in RSV-juice were also significant (11.5–18.0% at t7 and 24.8–31.3% at t14, at room temperature and 4 °C, respectively), while this effect was clearly delayed in γ-CD·RSV-juices, a fact that may have been promoted by the higher amount of RSV dissolved.

## 4. Conclusions

The present study describes the inclusion of RSV into γ-CD and the isolation of the inclusion complex as a solid product by co-dissolution, using food-safe solvents, and freeze-drying. The γ-CD·RSV complex was characterized by FT-IR, PXRD, and TGA and applied as a fortificant in fresh lemon juice. The solid-state analyses confirmed the formation of a true inclusion complex in which the cyclodextrin molecules are stacked into channels, with RSV molecules inside the cavity of the channels and a wide inter-channel space where water molecules are retained.

The amount of dissolved RSV in γ-CD·RSV-juice (45.8%) was increased compared to that of RSV-juice (5% of the target RSV concentration). Along storage time, the amount of RSV in RSV-juices and γ-CD·RSV decreased, independently of the storage temperature, possibly due to complex degradation/disintegration. The antioxidant capacity was similar between fortified juices and plain lemon juice, which may suggest that the antioxidant potential of RSV is low compared to that of other antioxidants in the juice and CA. Even so, the decrease of the antiradical activity towards ABTS^•+^ was less notorious in γ-CD·RSV-juices. Thus, the overall results indicate that the γ-CD·RSV inclusion complex is a suitable functional ingredient to increase the amount of RSV available in juices (still holding 15% after 28 days). This makes the complex a key element to improve the intake of RSV through the consumption of juices and, ultimately, enhancing its biological potential.

## Figures and Tables

**Table 1:** Physicochemical characteristics of formulated juices and citric acid solutions.

Formulation	pH	°Brix	Browning	Cloudiness	ᐃE
RSV-juice	2.52 ± 0.06 ^a^	8.7 ± 0.1 ^a^	0.19 ± 0.03 ^a^	0.14 ± 0.00 ^a^	5.56 ± 1.24 ^a^
γ-CD·RSV-juice	2.50 ± 0.04 ^a^	9.0 ± 0.1 ^a^	0.20 ± 0.02 ^a^	0.28 ± 0.01 ^ab^	6.00 ± 2.21 ^a^
RSV-CA	2.45 ± 0.33 ^a^	3.5 ± 0.1 ^b^	0.04 ± 0.01 ^b^	0.05 ± 0.01 ^a^	8.51 ± 1.76 ^a^
γ-CD·RSV-CA	2.56 ± 0.30 ^a^	3.7 ± 0.0 ^b^	0.05 ± 0.01 ^b^	0.51 ± 0.07 ^b^	8.40 ± 0.98 ^a^

Abbreviations: °Brix—Brix level; ᐃE—Total color difference; RSV-juice—juice with pure RSV; γ-CD·RSV-juice—juice with γ-CD·RSV inclusion complex; RSV-CA and γ-CD·RSV-CA—citric acid with pure RSV and γ-CD·RSV inclusion complex, respectively. Values presented as means ± standard deviation. Different superscript letters in the same column indicate significant differences (*p* < 0.05).

## Data Availability

The data that support the findings of this study are available within the article.
